# Miniaturization Potential of Additive-Manufactured 3D Mechatronic Integrated Device Components Produced by Stereolithography

**DOI:** 10.3390/mi16010016

**Published:** 2024-12-26

**Authors:** Niklas Piechulek, Lei Xu, Jan Fröhlich, Patrick Bründl, Jörg Franke

**Affiliations:** Institute for Factory Automation and Production Systems, Friedrich-Alexander-Universität Erlangen Nürnberg, Egerlandstr. 7-9, 91058 Erlangen, Germany; lei.x.xu@fau.de (L.X.);

**Keywords:** mechatronic integrated devices, MID, laser direct structuring, SLA, miniaturization

## Abstract

Three-dimensional Mechatronic Integrated Devices (3D-MIDs) combine mechanical and electrical functions, enabling significant component miniaturization and enhanced functionality. However, their application in high-temperature environments remains limited due to material challenges. Existing research highlights the thermal stability of ceramic substrates; yet, their reliability under high-stress and complex mechanical loading conditions remains a challenge. In this study, 3D-MID components were fabricated using stereolithography (SLA) 3D-printing technology, and the feasibility of circuit miniaturization on high-temperature-resistant resin substrates was explored. Additionally, the influence of laser parameters on resistance values was analyzed using the Response Surface Methodology (RSM). The results demonstrate that SLA 3D-printing achieves substrates with low surface roughness, enabling the precise formation of fine features. Electric circuits are successfully formed on substrates printed with resin mixed with Laser Direct Structuring (LDS) additives, following laser structuring and metallization processes, with a minimum conductor spacing of 150 µm. Furthermore, through the integration of through-holes (vias) and the use of smaller package chips, such as Ball Grid Array (BGA) and Quad Flat No-lead (QFN), the circuits achieve further miniaturization and establish reliable electrical connections via soldering. Taken together, our results demonstrate that thermoset plastics serve as substrates for 3D-MID components, broadening the application scope of 3D-MID technology and providing a framework for circuit miniaturization on SLA-printed substrates.

## 1. Introduction

Three-dimensional Mechatronic Integrated Devices (3D-MIDs) can effectively integrate mechanical, thermal, optical, and electrical functions due to their geometric design flexibility and the combination of selective structuring and metallization. As a result, they are used in various applications, such as automotive, medical technology, IT and telecommunications, and industrial automation [1]. Due to the unique three-dimensional structure and small batch production characteristics of 3D-MID components, the traditional injection molding process for manufacturing circuit carriers faces challenges such as high costs, limited design flexibility, long production cycles, and difficulty in handling complex geometries, all of which have become bottlenecks in the development of 3D-MID technology [2]. An innovative and rapid method for producing 3D-MID is the use of additive manufacturing technology (AM), which requires no molding tools. Additive manufacturing for 3D-MIDs not only compensates for the high initial cost of injection molds, but also offers greater design flexibility and faster production cycles [3]. Currently, extensive work is primarily focused on 3D-MIDs based on Fused Deposition Modeling (FDM) and Selective Laser Sintering (SLS) additive manufacturing technologies [2,4,5]. In order to further expand the application fields of 3D-MID, stereolithography (SLA) technology, which offers higher precision and better material properties, can be utilized. High-performance resins used in SLA, such as heat-resistant and high-strength resins, can enhance the performance of 3D-MID components, making them more suitable for demanding environments. The use of these resins extends the stability and reliability of 3D-MID components under high temperatures or mechanical loads [6,7,8].

In this work, circuit carriers were manufactured by mixing LDS additives with a heat-resistant resin and utilizing SLA 3D-printing technology. Taking temperature measurement systems as an example, after the LDS process and metallization, the circuit layout on the circuit carrier was successfully achieved. The introduction of vias and the use of smaller packaged components effectively realized circuit miniaturization.

To verify the feasibility of SLA-3D-MID components in practical applications, the relationship between laser structuring parameters and the resistance of circuits and vias was analyzed using the Response Surface Methodology (RSM). For soldering, vapor phase soldering (VPS) and reflow soldering were employed to solder the populated SLA-3D-MID components, ultimately producing SLA-3D-MID parts free of cracks.

## 2. Materials and Methods

### 2.1. Circuit Carrier Manufacturing

**Figure 1:**
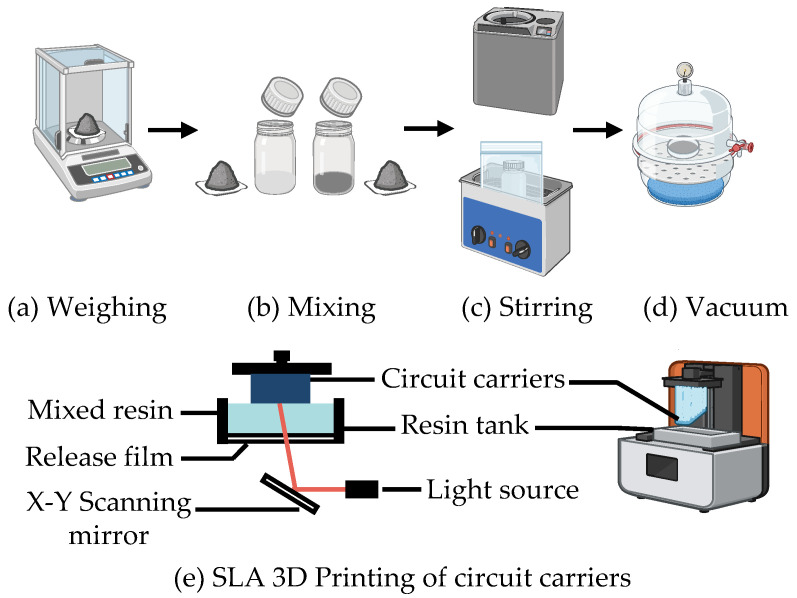
Resin mixing and circuit carrier printing.

The manufacturing of the circuit carriers, as shown in Figure 1a–d, utilized two different heat-resistant resins, each with LDS additives added as the printing material. The mixing ratios and mixing methods for each resin are provided in Table 1. To ensure that the LDS additives were fully mixed with the resin, the resin mixture was first stirred in a planetary mixer at 2000 rpm for 3 min, followed by mixing in an ultrasonic water bath for 10 min. For SLA printers, there are two main configurations: top-down and bottom-up [9]. The SLA 3D printer used in this work, the Mars 4 Ultra from ELEGOO, Shenzhen, China, operates with a bottom-up configuration, as shown in Figure 1e.

The first-layer exposure time and the normal layer exposure time for the mixed resin are shown in Table 2. Other parameter settings follow the default parameters of the original resin.

### 2.2. The Evaluation Criteria for Miniaturization

**Figure 2:**
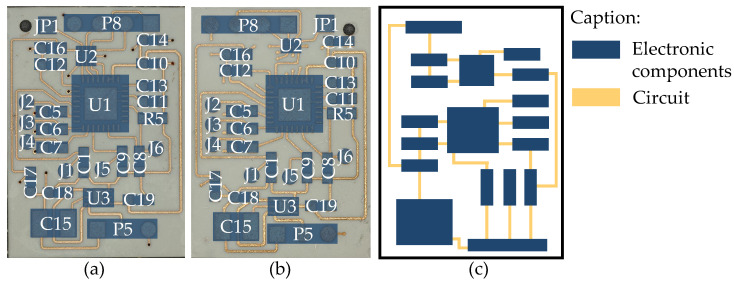
The circuit board with U2 in a (**a**) QFN package and (**b**) BGA package; (**c**) schematic diagram of the circuit board area.

In this work, the circuit design is shown in Figure 2a,b, with the only difference being the use of the U2 chip in different packages. The circuit is a temperature measurement system consisting of sensors connected to an AD converter, which requires a level shifter U2 and a voltage regulator U3. The dimensions of the circuit carrier are 18 mm × 25 mm. Equation (1) is used to evaluate the miniaturization, where AU represents the area utilization and S represents the area. The area of the circuit carrier cannot be infinitely reduced; it depends on the total area of the electronic components and the total area of the circuit. The closer the AU is to 1, the better the area utilization.

(1) $$AU=\frac{S_{\mathrm{Electronic}\;\mathrm{components}}+S_{\mathrm{Circuit}}}{S_{\mathrm{Circuit}\;\mathrm{carrier}}}$$

The schematic diagram of the areas of each part is shown in Figure 2c. In this work, only the area of the electronic components is considered. The areas of the individual electronic components are listed in Table 3.

### 2.3. LDS and Metallization

**Figure 3:**
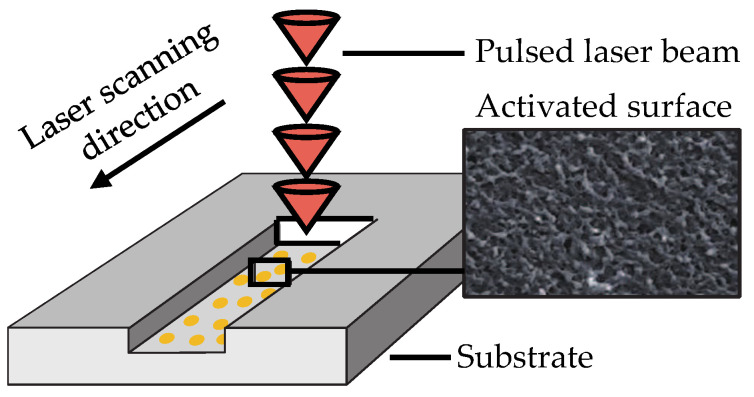
Schematic representation of laser activation [11].

As one of the representative 3D-MID methods, LDS, based on laser technology, is frequently used to create an electrically conductive metal layer on the surface of the modified thermoplastic polymer [5]. In this work, the resin used as the SLA 3D-printing material was a thermosetting plastic. Due to the additive in the resin (Figure 1b), it is possible to activate the surface, which was carried out as shown in Figure 3 using an infrared pulsed laser (1064 nm) with a pulse duration in the nanosecond range [10].

**Figure 4:**
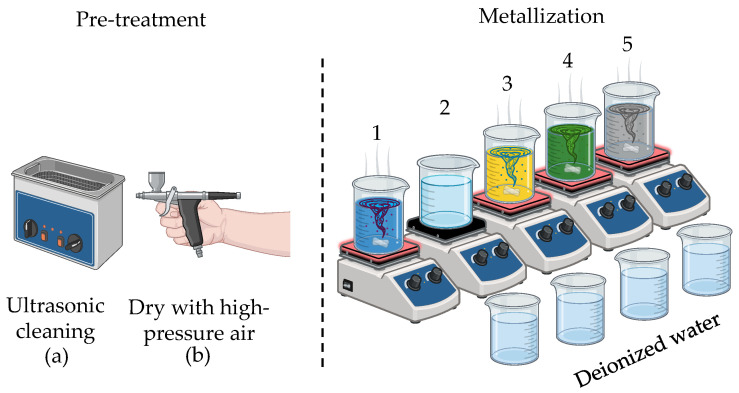
Schematic diagram of the metallization process.

The chemical electroless metallization process (Cupralux INI, Palladium Post Dip, Nickel HP, Gold HP) from Atotech, Berlin, Germany, was utilized in this work [10]. After laser structuring, the circuit carrier was metallized according to the process outlined in Figure 4.

Prior to the metallization of the 3D-MID components, the surface was pretreated with Enplate LDS Cleaner 300, MacDermid Alpha, Waterbury, USA solution to prevent excessive metallization caused by circuit miniaturization. After laser structuring, the component was cleaned in an isopropanol ultrasonic bath (Figure 4a) for 10 min, rinsed with deionized water, and dried using a high-pressure air blower (Figure 4b). The detailed steps of the metallization process are summarized in Table 4.

## 3. Results and Discussion

### 3.1. Post-Processing of SLA 3D-Printed Circuit Carrier

**Figure 5:**
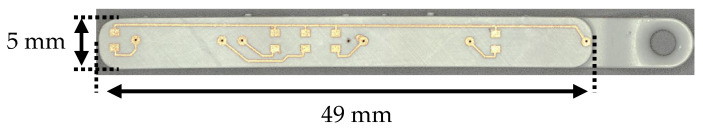
Schematic diagram of sensor circuit carrier dimensions.

The dimensions of the sensor circuit carrier are shown in Figure 5. After SLA 3D-printing, it is first cleaned with isopropanol/soap water, then undergoes UV curing (TR300 for 30 min, UW for 10 min), and is finally placed in an oven at 200 °C for 1 h.

**Figure 6:**
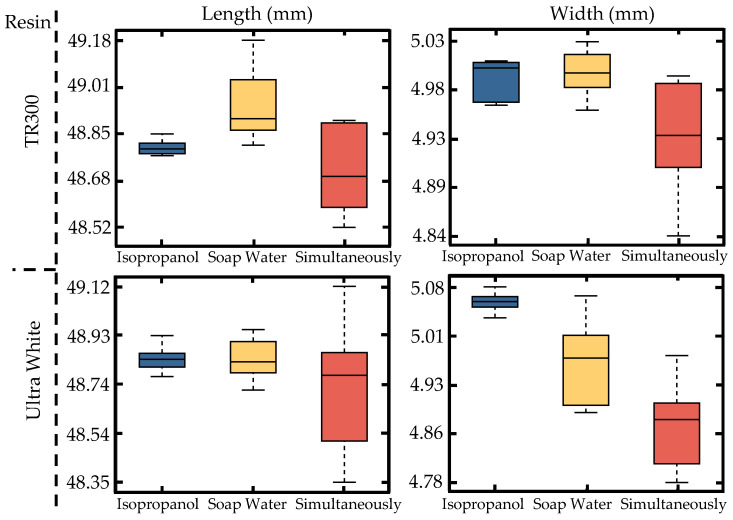
Box plot of the impact of different post-processing methods on circuit carrier dimensions.

The experiment includes three control groups, with cleaning liquids for the sensor circuit carrier set as isopropanol, soap water, and a combination of soap water cleaning with simultaneous UV curing (simultaneously). After post-processing, the dimensions of each group are measured, with the results shown in Figure 6.

From the box plot in Figure 6, it can be concluded that the treatment of resin samples with isopropanol (shown in blue in Figure 6) is the most effective, as it shows the least variation and the highest stability. The simultaneous cleaning and curing process shows greater variation due to the scattering of the UV light as it enters the liquid, preventing it from accurately reaching the surface of the part to be cured.

### 3.2. Surface Quality of the Substrate

**Figure 7:**
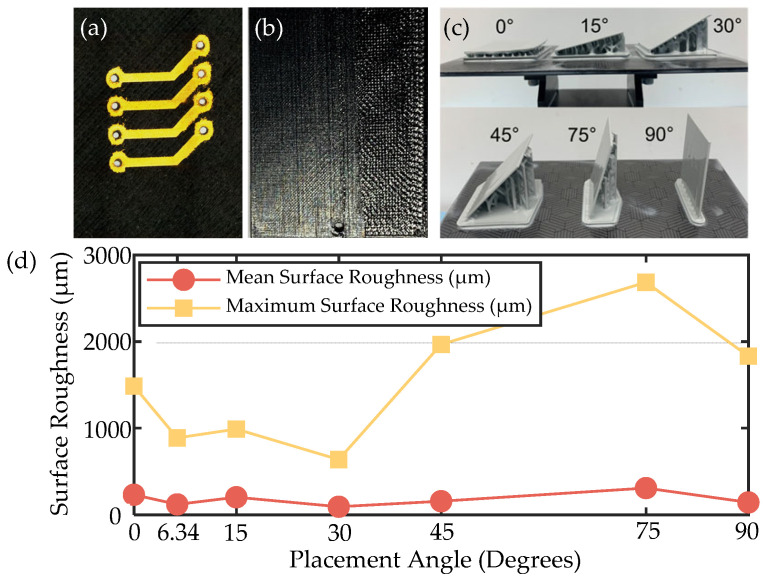
(**a**) MID components with FDM-printed substrate as circuit carriers, (**b**) FDM-printed substrate, (**c**) SLA-printed substrate placed at different angles, (**d**) line chart of surface roughness for SLA substrates.

The surface roughness of the substrate significantly influences the roughness of the final metallized layer. As shown in Figure 7a,b, the substrate surface fabricated by FDM printing exhibits pronounced texturing due to the stair-stepping effect. Excessive surface roughness can lead to the formation of large gaps during metallization, which, in turn, induces or propagates microcracks. For wider trace widths, crack propagation requires more cycles to result in complete fracture, thus having an insignificant impact on Mean Time to Failure (MTTF). However, as circuits are further miniaturized, the presence of microcracks can lead to fractures under fewer thermal cycles or mechanical loads, thereby increasing conductor resistance and degrading electrical performance [12,13].

SLA 3D-printing significantly outperforms FDM due to its superior surface quality. As shown in Figure 7c,d, while different placement angles can affect the substrate surface quality, no clear trend is observed in Mean Surface Roughness. The low surface roughness facilitates circuit miniaturization and supports the assembly of electronic components [14].

### 3.3. The Miniaturization of Circuits

The miniaturization of circuits includes the following three aspects: the introduction of vias, the reduction of spacing between traces, and chips with smaller packaging specifications.

#### 3.3.1. Via Holes

**Figure 8:**
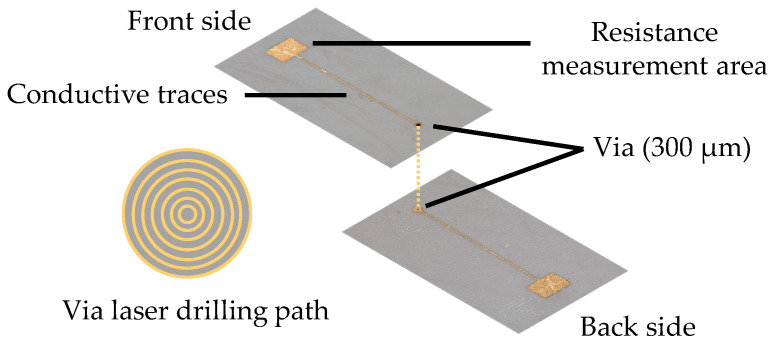
Schematic diagram of a via hole in the circuit carrier.

Via refers to the vertical electrical connection between two conductor layers [15]. Structures as shown in Figure 8 are created on both the front and back sides of the printed circuit carrier using laser drilling with the Fusion3D 1100, LPKF, Garbsen, Germany.

**Figure 9:**
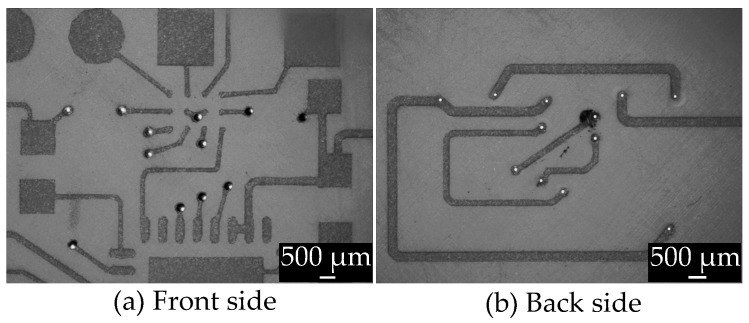
Microscopic close-up of via holes in the circuit.

A specific limitation in 3D-MID design is that the layer structure can only support a maximum of two layers and lacks a ground plane, requiring all grounding to be connected through routing [3]. Therefore, vias are even more essential to route connections on both sides of the 3D-MID structure. The circuit is arranged on the front side of the circuit carriers in Figure 9a and the back side in Figure 9b, with connections established through vias.

**Figure 10:**
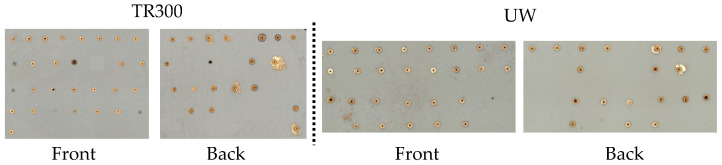
Experimental substrate with via holes for TR300 and UW.

The laser parameters for via holes are investigated using Response Surface Methodology (RSM) and screening experimental design. The RSM method uses a Box–Behnken design. The factors and levels are shown in Table 5. The 29 experiments for each of the two resins are shown on the substrate in Figure 10.

Figure 10 illustrates that different combinations of laser parameters have varied impacts on the surface around the vias after metallization. During the drilling process, smoke can be generated, leading to unintended metallization around the via, which may cause incorrect circuit connections when the vias are part of a circuit, as shown in Figure 9. Additionally, when the operating power is set to 20%, it fails to penetrate the 1 mm thick substrate, preventing the formation of vias.

After metallization, the resistance values of the vias were measured. An ANOVA analysis was performed on the resistance values for TR300, with the results shown in Table 6. The resistance values on UW are consistently 0.2 Ω, showing no statistical significance, and there is no relationship between resistance values and laser parameters.

**Figure 11:**
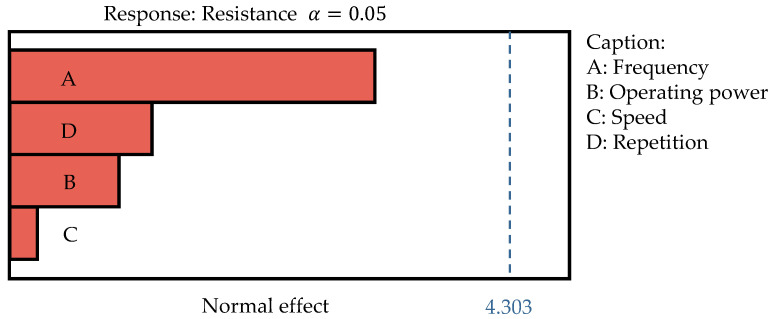
Pareto chart analysis of via holes’ resistances on TR300 substrate.

A *p*-value less than 0.05 indicates that the model terms are significant. However, the significant *p*-value for the lack-of-fit term (*p* = 0.0254 < 0.05) indicates that the model does not adequately fit the data. To further confirm whether the four factors impact the resistance values, a screening experimental design (totaling 13 experiments) is used, and the results are evaluated using the Pareto chart shown in Figure 11.

The blue dashed line in Figure 11 represents the significance threshold (α=0.05), with a value of 4.303. Factors exceeding this threshold are considered to have a significant impact on the response variable (resistance). In this case, none of the four factors surpasses the significance threshold, indicating that they do not affect resistance.

**Figure 12:**
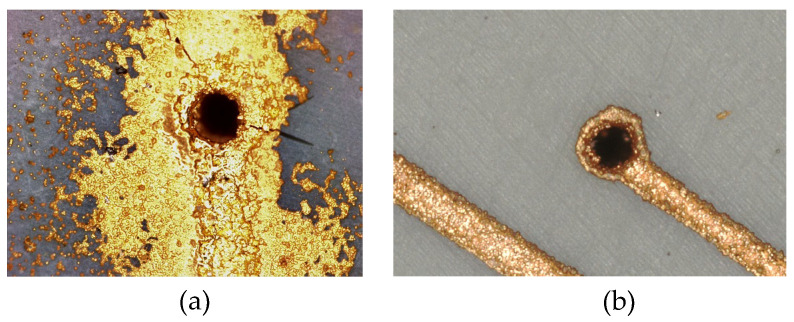
(**a**) Via hole showing excessive metallization around the surrounding area; (**b**) via hole showing no excessive metallization around the surrounding area.

Based on the above analysis, it can be concluded that when laser drilling is performed on these two resins using the LPKF Fusion3D 1100, the laser parameters do not influence the resistance of the subsequently metallized via holes. Although the laser parameters do not affect the resistance values of the via holes, different frequencies and operating power levels impact the surface surrounding the via holes. As shown in Figure 12a, when a significant amount of smoke is generated during drilling, it leads to excessive metallization around the via holes after metallization. The laser parameters used in the experiment are selected to ensure that, as shown in Figure 12b, the surface around the via holes does not exhibit excessive metallization. The laser parameters are provided in Table 7.

#### 3.3.2. Circuit

**Figure 13:**
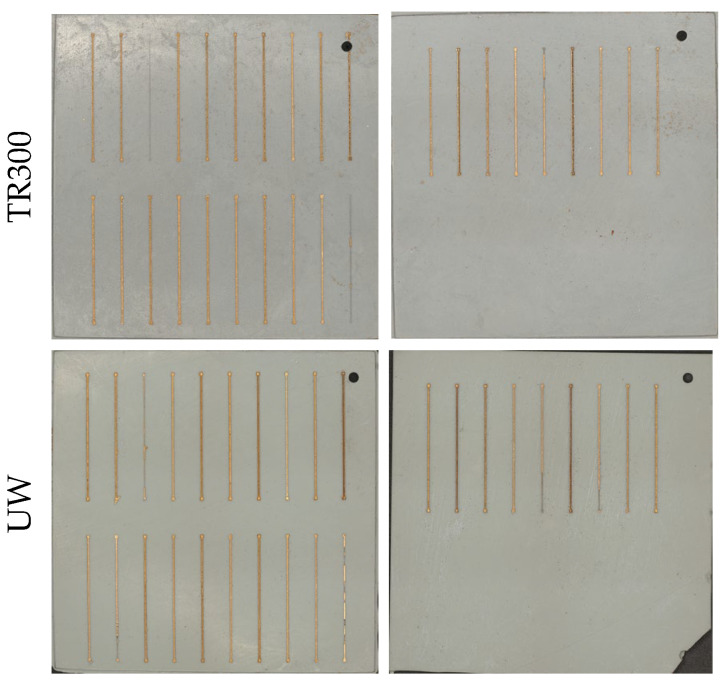
Experimental substrate with traces for TR300 and UW.

The laser parameters for circuits are determined using an RSM experimental design (Box–Behnken). The factors and levels are shown in Table 8. The 29 experiments for each of the two substrates are shown in Figure 13.

**Figure 14:**
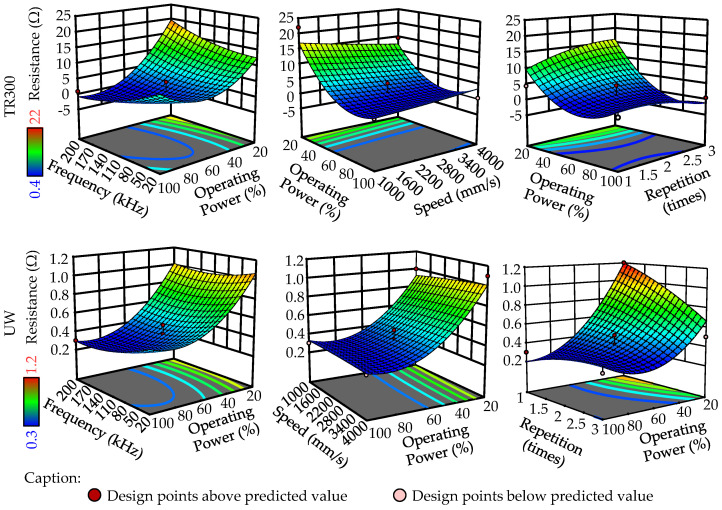
3D response surface plot of resistance for different factors between TR300 and UW.

After measuring the resistance values of all traces, the results were analyzed using ANOVA, with the findings listed in Table 9. In the ANOVA analysis, the Model term being significant and the Lack of Fit term being non-significant indicate that the model fits the experimental data well. Operating power is a significant factor for both types of substrates (*p*-value < 0.05). Based on this reason, the choice of laser parameters for the two different materials depends on the impact of operating power and the combination of the other three factors on resistance values. The three-dimensional surface diagram of the response resistance values for the two materials is shown in Figure 14.

**Figure 15:**
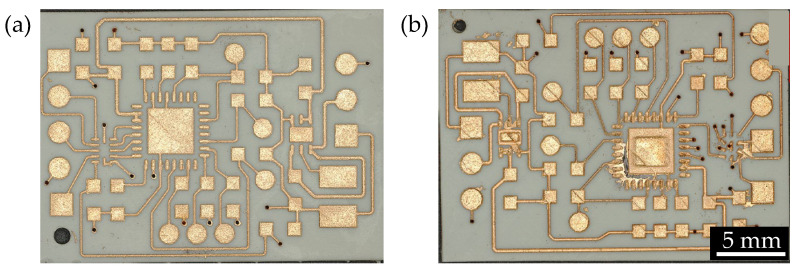
(**a**) Optimized circuit and (**b**) over-metallized circuit.

When the operating power reaches 20%, the resistance value is at its maximum. If the operating power is too low, the surface roughness achieved by laser structuring is insufficient, which compromises the subsequent metallization process. As shown in Figure 13, the deposition of metal is uneven, leading to an increase in resistance values. However, an excessively high operating power can lead to over-metallization of the surface, as illustrated in Figure 15b.

Negative resistance values are observed in Figure 14 due to the RSM being an approximate model (all negative resistance values are disregarded in this experiment). The laser parameters for both materials are listed in Table 10.

For the UW resin, the experimental setup does not select the laser parameter combination that minimizes resistance. This is because over-metallization occurs on the substrate surface following metallization when the frequency is set at 200 kHz and operating power at 80% or 100%, as shown in Figure 15b. Using the laser parameters listed in Table 10, an optimized circuit as depicted in Figure 15a can be achieved.

**Figure 16:**
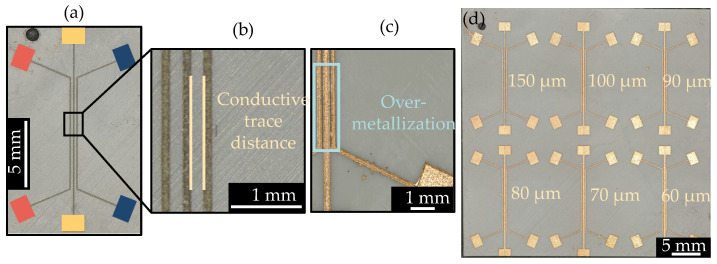
(**a**) Schematic diagram of the experimental structure for a single minimum circuit spacing; (**b**) Schematic diagram of conductive trace distance; (**c**) Schematic diagram of via metallization; (**d**) Experimental substrate for minimum circuit spacing.

The test structure for the minimum distance between conductive traces is shown in Figure 16. A total of six distances are set, as shown in Figure 16d. The distances are achieved by increasing the width of the middle conductive trace, as shown in Figure 16b. The measurement rule in the experiment is as follows: the measurement area of same-color blocks, as shown in Figure 16a, should detect resistance, while the measurement area of different-color blocks should show no resistance. If resistance is detected in the measurement area of different-color blocks, it is determined that over-metallization (Figure 16c) has occurred for the corresponding conductive trace distance after metallization. In all experiments, only when the distance between the conductive traces is 150 µm (approximately 6 mil) does the measurement rule hold true.

#### 3.3.3. Chips with Different Packaging

**Figure 17:**
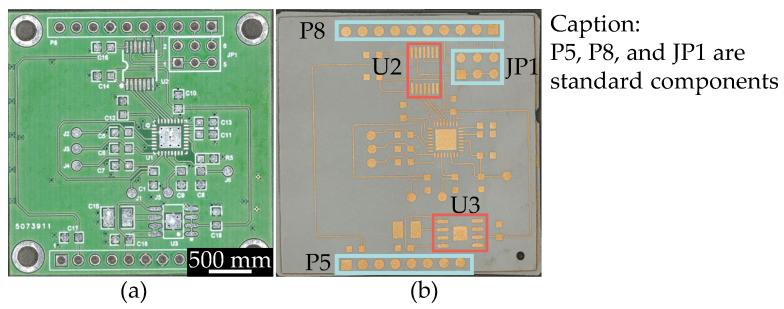
The circuit on the (**a**) traditional circuit board and (**b**) SLA-printed circuit carrier.

After conducting circuit design in Altium Designer, the manufacturability of the original circuit was first verified on a traditional circuit board as shown in Figure 17a. Then, the circuit was created using LDS on the SLA-printed circuit carrier as shown in Figure 17b, followed by metallization. The dimensions of both the PCB and the SLA-circuit carrier are 40 mm by 40 mm. The model and package type of U2 and U3 in the red area of Figure 17b are listed in Table 11.

**Figure 18:**
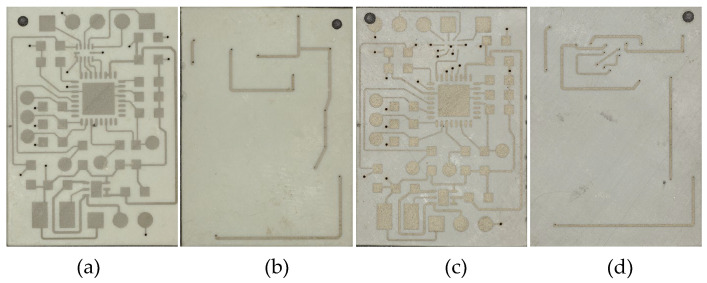
The front (**a**) and back (**b**) views of the miniaturized circuit using a QFN package (UW), and the front (**c**) and back (**d**) views of the miniaturized circuit using a BGA package (TR300).

To further reduce the size of the circuits, U2 and U3 with smaller package sizes are used. The layout is redesigned in Altium Designer 24.2.2, and the miniaturized circuit is placed on an SLA-circuit carrier with dimensions of 25 mm by 18 mm, as shown in Figure 18 (before metallization).

Conductive traces and via holes were created using the laser parameters from Table 7 and Table 10. The width of the conductive traces was 6 mil, and the minimum spacing between the edges of the conductive traces, as well as between the conductive traces and other components, was also 6 mil.

**Figure 19:**
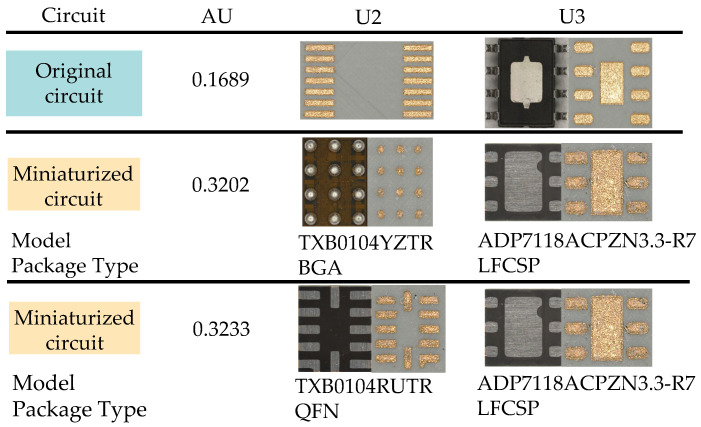
AU for circuit miniaturization and chip packages in original and miniaturized circuits.

All chip packages used in the original circuit and the miniaturized circuit are shown in Figure 19. The package models and types used in the original circuit are listed in Table 11.

### 3.4. Assembling and Soldering

**Figure 20:**
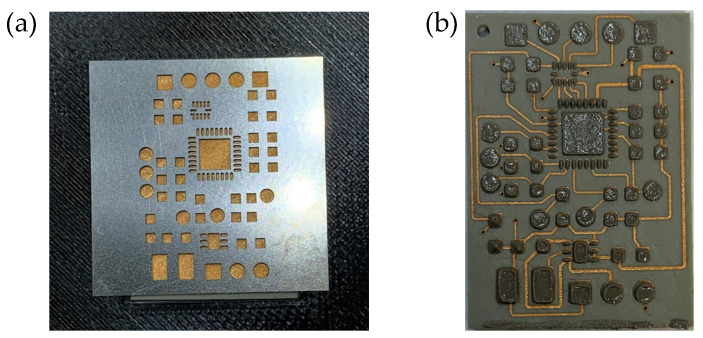
(**a**) Solder paste stencil; (**b**) circuit board with applied solder paste.

Before assembling and soldering, the metallized circuit board needs to be placed in an oven at 175 °C for one hour to bake. The solder paste is applied to the metallized circuit board using a stencil, as shown in Figure 20a. The placement of electronic components is performed manually with the aid of a machine.

In this work, two different soldering methods were used, namely condensation soldering and convection soldering. Table 12 lists the soldering methods used for the two materials.

**Figure 21:**
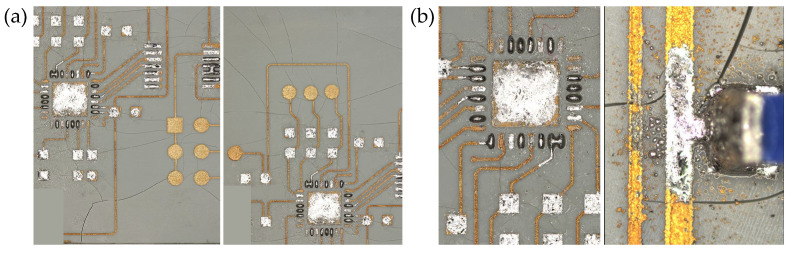
(**a**) Cracks on the circuit board (Material: UW), (**b**) flow of solder along the circuit.

**Figure 22:**
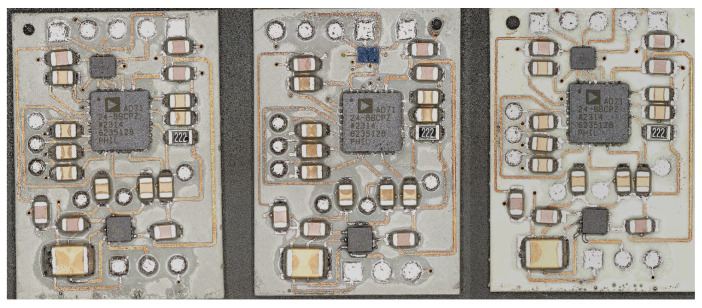
The complete circuit board.

Using condensation soldering to solder the circuit board made by UW can cause cracking, as shown in Figure 21a. Additionally, after soldering, the solder was observed to have a tendency to flow along the circuit, as shown in Figure 21b. The circuit board after soldering is shown in Figure 22.

The difference in the coefficient of thermal expansion (CTE) between the substrate and the metallization layer can lead to stress concentration, potentially causing cracks and reducing the adhesion strength of the metallization layer. During reflow soldering or vapor phase soldering processes, the rapid temperature changes induce significant stresses due to thermal expansion mismatches between materials. These stresses primarily originate from two factors: (1) the thermal expansion mismatch caused by the differing CTEs of the substrate and the metallization layer, and (2) the mechanical coupling effect between the metallization layer and the substrate. The accumulation of these stresses may further result in component self-alignment phenomena and positional drift, thereby affecting the quality and reliability of the soldering process [12,13,16].

**Figure 23:**
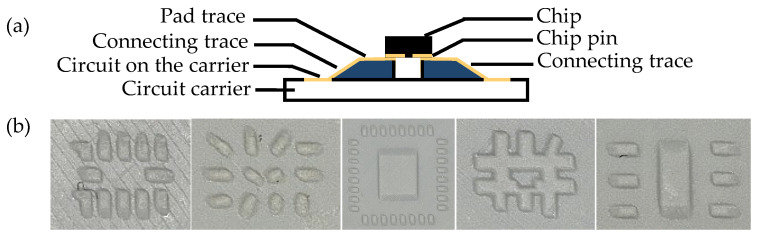
(**a**) Schematic diagram of 3D pads, (**b**) real-life images of 3D pads for different chips.

**Figure 24:**
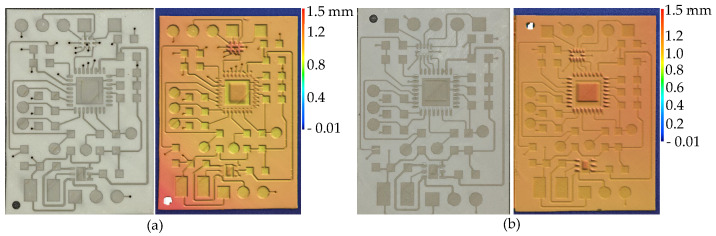
Using 3D pads on circuits with (**a**) BGA/(**b**) QFN packaged chips.

In 3D-MID technology, the circuit carrier lacks the solder mask layer found on traditional PCBs. This characteristic poses a particular challenge for mounting chips with a narrow pin spacing. To accurately place these chips, this work utilized 3D pads, as shown in Figure 23. By elevating the pads, mis-soldering between the chip pins was isolated. The 3D pads in the complete circuit are shown in Figure 24.

For BGA-packaged chips, in addition to using 3D pads, this work also experimented with Anisotropic Conductive Adhesive (specifically 3M™ Electrically Conductive Adhesive Transfer Tape 9703, 3M, Maplewood, NJ, USA). This adhesive conducts electricity only in the Z-axis direction, making it highly suitable for chips with dense pin configurations. The conductive materials inside the Anisotropic Conductive Adhesive are sparsely distributed, preventing conductivity in the X and Y axis directions.

### 3.5. Test

**Figure 25:**
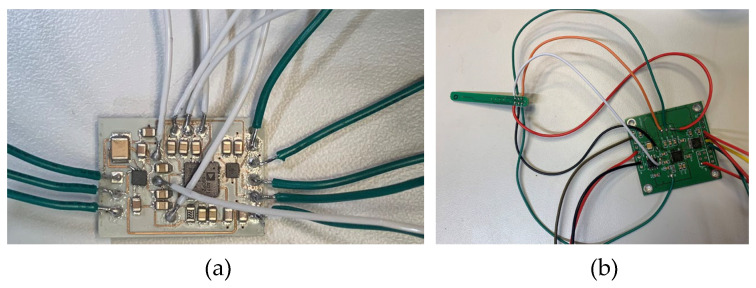
(**a**) Wire connections on the SLA-MID circuit board, (**b**) validation circuit (standard PCB board).

In this work, the circuit was based on EVAL-CN0391-ARDZ, Analog Devices, Norwood, USA. The circuit board in Figure 22 needs to be connected with wires to the sensor circuit carrier shown in Figure 5, as illustrated in Figure 25. Once connected, this system runs on an Arduino.

**Figure 26:**
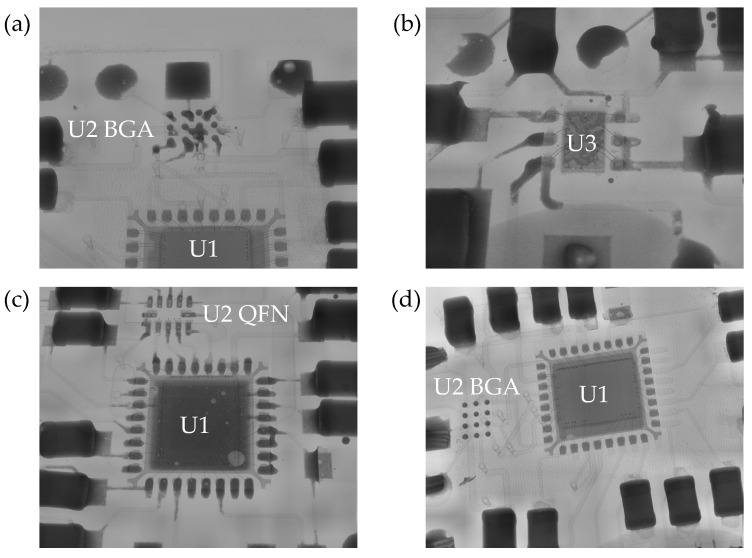
(**a**–**d**) X-ray scan of the resin circuit board.

In all tests, the temperature was not measured correctly, and the circuit exhibited smoking due to a short circuit. The validation circuit shown in Figure 25b operates correctly and retrieves temperature readings, thus ruling out any flaws in the circuit design. To determine the location of the short circuit on the resin circuit boards, all resin circuit boards are subjected to X-ray scanning. The results are shown in Figure 26.

From Figure 26, it can be seen that after soldering, there is no excess solder between the pins of chips U1, U2 (QFN), and U3. However, in Figure 26a, the pads of chip U2 (BGA) have excess solder, which affects the quality of the soldering. This excess solder may cause a short circuit. Three-dimensional pads can partially mitigate the issue of short circuits caused by the dense arrangement of chip pins after soldering. However, for electronic components in small package formats, the contact area of the solder during the wetting process is extremely small and unevenly distributed. Additionally, achieving precise alignment of electronic components during the assembly process and controlling circuit alignment during the LDS process remain challenging.

## 4. Conclusions and Outlook

In summary, this study explored the miniaturization of 3D-MID components facilitated by SLA 3D-printing technology. The focus on circuit miniaturization included the introduction of vias, reduction of trace spacing, and implementation of chips with smaller packaging, such as BGA and QFN. An RSM experimental design and screening experiments optimized laser parameters, revealing that excessive operating power leads to over-metalization. Pre-treatment with Enplate LDS Cleaner 300 solution effectively reduced these over-metalization effects. Notably, the width of the circuit lines is not a primary target for miniaturization; instead, the arrangement of electronic components, adoption of smaller chip packages, and strategic circuit configurations are crucial in determining the overall size of the circuit carriers. Furthermore, a minimum spacing of 150 µm between circuits is established. Consequently, the components based on SLA 3D-printing technology for 3D-MID show immense potential for advancing miniaturization, which not only supports the integration of complex circuits and precise wiring but also significantly enhances the development of miniature electronic devices and systems. Building on these findings, future research will focus on further enhancing the adhesion between the substrate and the metallization layer, as well as mitigating any remaining challenges related to cracks caused by differences in CTE, to improve the reliability of 3D-MID components.

## Figures and Tables

**Table 1:** The mixing ratio and stirring method of the resin.

Resin	LDS Additives	Stirring Method
Phrozen TR300 (TR300)	3%	Planetary Mixer and Ultrasonic Water Bath
Siraya Tech Ultra White (UW)	3%	Planetary Mixer and Ultrasonic Water Bath

**Table 2:** First-layer and normal-layer exposure times for mixed resins.

Resin	First-Layer (s)	Normal Layer (s)
TR300	75	1.2
UW	55	2.5

**Table 3:** The names and areas of the electronic components.

Component	Name	Area (mm^2^)
C1, C5–C14, C16–C19	Capacitance	3
C15	Capacitance	11.9
J1–J6	Measurement Point	2.7
R5	Resistance	3
JP1/JP1 Standard Component	Pin header	2.7/26.6
P5/P5 Standard Component	Pin header	11/32.04
P8/P8 Standard Component	Pin header	16/40.4
U1	Chip	31.3
U2 BGA/U2 QFN	Chip	2/3.42
U2 TSSOP (Original circuit)	Chip	31.4
U3	Chip	5
U3 SOIC (Original circuit)	Chip	32.5

**Table 4:** Summary of metallization process operating steps.

Number in Figure 4	Solution	Temperature (°C)	Time
1	Copper	55	60 min
2	Acid	-	30 s
3	Palladium	-	1 min
4	Nickel	62	60 min
5	Gold	90	30 min

**Table 5:** Factors and levels in the RSM method (via).

Factor	Level
Frequency	20, 110, 200 (kHz)
Operating power	20, 60, 100 (%)
Speed	1000, 2500, 4000 (mm/s)
Repetition	750, 825, 900 (times)

**Table 6:** ANOVA analysis of resistance values of via holes on TR300 substrate (excerpt).

Factor	Sum of Squares	df	Mean Square	F-Value	*p*-Value	
Model	99.72	10	9.97	794.19	<0.0001	significant
Frequency	0.0023	1	0.0023	0.1820	0.6845	
Operating power	0.0310	1	0.0310	2.47	0.1670	
Speed	0.0023	1	0.0023	0.1820	0.6845	
Repetition	38.48	1	38.48	3064.79	<0.0001	
…	…	…	…	…	…	…
Lack of Fit	0.0633	2	0.0317	10.56	0.0254	significant

**Table 7:** Laser parameters for via holes on both resin types.

Frequency (kHz)	Operating Power (W)	Speed (mm/s)	Repetition (Times)
20	3.47 (100%)	1500	900

**Table 8:** Factors and levels in the RSM method (circuit).

Factor	Level
Frequency	20, 110, 200 (kHz)
Operating Power	20, 60, 100 (%)
Speed	1000, 2500, 4000 (mm/s)
Repetition	1, 2, 3 (times)

**Table 9:** ANOVA analysis of resistance values of circuits on TR300/UW (excerpt).

Factor	Sum of Squares	df	Mean Square	F-Value	*p*-Value	
Model	627.22	14	44.8	3.93	0.0113	significant
Model	1.43	14	0.1019	11.08	<0.0001	significant
Frequency	1.62	1	1.62	0.1423	0.7126	
Frequency	0.0313	1	0.0313	3.40	0.0901	
Operating Power	347.98	1	347.98	30.56	0.0001	significant
Operating Power	0.9425	1	0.9425	102.50	<0.0001	significant
Speed	2.80	1	2.80	0.2462	0.6288	
Speed	0.0033	1	0.0033	0.3625	0.5583	
Repetition	8.17	1	8.17	0.7172	0.4136	
Repetition	0.1008	1	0.1008	10.97	0.0062	
…	…	…	…	…	…	…
Lack of Fit	124.77	8	15.6	5.25	0.0633	not significant
Lack of Fit	0.0903	8	0.0113	2.26	0.2248	not significant

**Table 10:** Laser parameters for TR300 and UW.

Frequency (kHz)	Operating Power (W)	Speed (mm/s)	Repetition (times)
110	9.67 (60%)	2500	1
110	9.67 (60%)	2500	1

**Table 11:** Model and package type of U2 and U3 in the original circuit.

Chip	Model	Package Type
U2	TXB0104PWR	TSSOP
U3	ADP7156ARDZ3.0R7	SOIC

**Table 12:** Soldering methods and principles.

Machine	Principle	Material
IBL LC 280, IBL-Löttechnik GmbH, Königsee, Germany	Condensation Soldering	TR300
ERSA Hotflow 2/14, ERSA GmbH, Wertheim, Germany	Convection Soldering	TR300 and UW

## Data Availability

The original contributions presented in this study are included in the article. Further inquiries can be directed to the corresponding author.
